# Effectiveness and safety of high-voltage pulsed radiofrequency to treat patients with primary trigeminal neuralgia: a multicenter, randomized, double-blind, controlled study

**DOI:** 10.1186/s10194-023-01629-7

**Published:** 2023-07-18

**Authors:** Yitong Jia, Hao Cheng, Niti Shrestha, Hao Ren, Chunmei Zhao, Kunpeng Feng, Fang Luo

**Affiliations:** 1 Department of Anesthesiology, Xuanwu Hospital, Capital Medical University, Beijing, China; 2 Department of Anesthesiology, Beijing Ditan Hospital, Capital Medical University, Beijing, China; 3 Department of Pain Management, Beijing Tiantan Hospital, Capital Medical University, Beijing, China

**Keywords:** Trigeminal neuralgia, Effectiveness, Safety, High-voltage, Pulsed radiofrequency

## Abstract

**Background:**

Trigeminal neuralgia (TN) is a debilitating pain disorder that still lacks an ideal treatment option. Pulsed radiofrequency (PRF), especially with high output voltage, is a novel and minimally invasive technique. PRF is regarded a promising treatment option for TN patients who respond poorly to medical treatment; however, the available evidence still lacks high quality randomized controlled trials (RCTs). Our study aimed to evaluate the long-term (1 year and 2 years) effects and safety of high-voltage PRF in primary TN patients and provide stronger evidence for TN treatment options.

**Methods:**

We performed a multicenter, double-blind, RCT in adults (aged 18–75 years) with primary TN who responded poorly to drug therapy or were unable to tolerate the side effects of drug. Eligible participants were randomly assigned (1:1) to receive either high voltage PRF or nerve block with steroid and local anesthetic drugs. The primary endpoint was the 1-year response rate. This trial has been registered in the clinicaltrials.gov website (registration number: NCT03131466).

**Results:**

One hundred and sixty-two patients were screened for enrollment between April 28th,2017 and September1st, 2019, among whom, 28 were excluded. One hundred and thirty-four participants were randomly assigned to either receive high voltage PRF (*n* = 67) or nerve block (*n* = 67). The proportion of patients with a positive response at 1-year after the procedure in the PRF group was significantly higher than that in the nerve block group in the intention-to-treat population (73.1% vs. 32.8%, *p* < 0.001). There was no difference between groups in the incidence of adverse events.

**Conclusions:**

Our findings support that high voltage PRF could be a preferred interventional choice prior to receiving more invasive surgical treatment or neuro-destructive treatment for TN patients who have poor responses to medical treatment.

**Trial registration:**

Our study has been registered at ClinicalTrials.gov (trial registration number: NCT03131466).

**Supplementary Information:**

The online version contains supplementary material available at 10.1186/s10194-023-01629-7.

## Background

Trigeminal neuralgia (TN) is a debilitating pain disorder characterized by paroxysmal stabbing pain in the facial region innervated by the trigeminal nerve. In most severe cases, patients become fearful of basic daily activities such as talking, eating, drinking, or touching the face [1, 2]. Epidemiological studies have reported that the incidence of TN is approximately 4 to 28.9/100,000 persons worldwide [3]. TN patients usually present with increased risk of anxiety, depression and poor sleep which could cause poor quality of life (QoL) and severe psychological disturbances [4]. The first-choice treatment for TN is anticonvulsants. However, the recurrence rate with drug therapy is as high as 25–50% within 12 weeks [5]. Furthermore, anticonvulsant drugs are associated with side effects such as dizziness, diplopia, ataxia, and elevated aminotransferase levels [6] which limited its clinical application. A recent study also suggests an alternative pharmaceutical treatment option, Calcitonin gene-related peptide (CGRP) modulator biological therapies such as the ones used in the treatment of migraine headache, for the treatment on TN [7], based on reports of elevated CGRP levels in the cerebrospinal fluid and blood during acute episodes of TN. However, since the literature only includes case reports and observational studies, this may be considered a limitation of the study and their suggestions must be acknowledged with caution.

Silvia et al. [8], provide excellent insights regarding the treatment of TN and highlight the need for improvement and updates in the management of TN; as an estimated 50% of patients fail to achieve complete pain relief with medico/pharmacological options and ultimately opt for surgical interventions. Meta-analyses have reported that microvascular decompression (MVD) was the most efficacious surgical treatment for classical TN caused by trigeminal nerve compression by a branch of the basilar artery which provides pain relief for 4–5 years in 61 to 80% patients [9]. But at the same time, MVD is associated with several major postoperative complications (such as cerebrospinal fluid leakage in 2.0%, brain-stem infarctions or hematomas in 0.6%, meningitis in 0.4% or even death in 0.3%) [4, 10]. Other invasive procedures including radiofrequency thermocoagulation [11], glycerol injection [12], balloon compression (BC) [13, 14] may be additional treatment options for TN patients who respond poorly to medical therapy. These percutaneous interventions damage the trigeminal ganglion in the Meckel’s cave or exiting branches of the ganglion at the base of the skull and can achieve immediate and long-term pain relief. However, adverse effects such as corneal deafferentation, resultant keratitis, long-lasting severe trigeminal sensory deficits, etc. caused by these procedures must not be ignored [9]. Stereotactic radiosurgery is a non-invasive, neuro-destructive technique that focuses the radiation beams on the trigeminal root before it enters the pons [9]. Studies show that, 24 to 71% of patients could achieve pain relief for 1 to 2 years following gamma knife treatment. However, this stereotactic radiosurgery procedure does not attain immediate pain relief but would take 6 to 8 weeks to develop pain-relieving effect. Moreover, the incidence of facial numbness following gamma knife treatment is up to 16% [15]. Since all available invasive options have their drawbacks, there is an overwhelming need for finding a safe, minimally invasive treatment option for TN.

Nerve block with local anesthetic drugs is a well-accepted and tolerated procedure for the diagnosis of TN. It has also been proved to be effective in the treatment of TN with minimal risks [16]. However, the response rate is only 34.3%, 1 day after the block and the recurrence rate is approximately 58% within 72 weeks, despite the application of high concentration lidocaine [17]. The addition of steroid could achieve prolonged pain relief. It is reported that the effective rate of nerve block with local anesthetic and steroid at 1 month postoperatively was 25%, and the duration of effective pain relief was 16.2 ± 12.7 weeks after the first block [18]. But most TN patients who underwent nerve block usually need multiple repeated interventions which may raise concerns such as puncture related risks, local anesthetic drugs and steroid-associated side effects [19]. In recent years, pulsed radiofrequency (PRF), a novel and minimally invasive technique has been shown to be a promising treatment option for TN. This micro-destructive procedure consists of a continuous high energy current of 20 ms and an intermission period of 480 ms. Therefore, the heat of the electrode tip can be dissipated during this interval, the temperature will not exceed 42 °C and the induced target tissue injury caused by this procedure is minimal [20–22]. Nevertheless, Elawamy et al. [23] reported that the long-term outcomes of PRF was not satisfactory. Increasing the output voltage of PRF may further improve the treatment efficacy. Our randomized controlled study demonstrated that the 1-year effective rate of high-voltage PRF (69%) was significantly higher than that of a standard-voltage group(19%) (p < 0.05) for refractory TN patients who respond poorly to both medical treatment and nerve block with local anesthetic and steroid [24]. In 2016, our team conducted a prospective clinical research which reported that the 2-year response rate of high-voltage PRF for the treatment of medically unresponsive TN patients was up to 78.6% [25]. However, this was a single center study with a small sample size. Multicentric randomized controlled trials with larger sample size are needed to further evaluate the effectiveness and safety of high voltage PRF for TN therapy. To provide stronger evidence, a sham PRF treatment without radiofrequency energy output as a control should be used. But in consideration of ethical issues, our study utilized nerve blocks with local anesthetic and steroid as a treatment technique in the control group.

In the present study, we conducted a prospective, multicenter, randomized, double-blind, controlled clinical trial to assess the long-term effects and safety of high-voltage PRF for primary TN patients who failed to respond to drug treatment.

## Methods

### Study patients

In accordance with the ethical principles of the Declaration of Helsinki and Good Clinical Practice (GCP) guidelines, this prospective, multicenter, double-blind, randomized controlled clinical trial was conducted from April 2017 and September 2021. Our study has been approved by the Institutional Review Board (IRB) of Beijing Tiantan Hospital (Approval Number: KY 2017-004-01) and was registered at ClinicalTrials.gov (trial registration number: NCT03131466). All patients signed written informed consent. We enrolled primary TN patients who responded poorly to drug treatment and were scheduled for receiving high-voltage PRF or nerve block with local anesthetic and steroid in Beijing Tiantan Hospital, Beijing Friendship Hospital and Beijing Ditan Hospital. All patients signed written informed consents.

### Inclusion criteria

The inclusion criteria were as follows: age 18 to 75 years; primary TN patients who meet the criteria of International Classification of Headache Disorders 3rd Edition (beta version) (ICHD-3beta) [26] and ICHD-3 [1] and responded poorly to drug treatment or were unable to tolerate the side effects of drug; Barrow Neurological Institute(BNI) pain intensity score of IV-V; be supposed to undergo neurosurgical intervention according to TN treatment guidelines [2]; signed informed consent.

### Exclusion criteria

The exclusion criteria included coagulation disorders or bleeding disorders; severe cardiopulmonary dysfunction; allergy to local anesthetic drugs or steroids; patients taking analgesic drugs other than carbamazepine; history of mental illness; history of narcotic drug abuse; history of invasive treatments such as radiofrequency thermocoagulation, chemical ablation, balloon compression surgery, microvascular decompression, gamma knife, etc.

### Randomization and sequence generation

A total of 162 patients were initially screened for eligibility and 28 patients were excluded on account of refusal from participation, or for not meeting the inclusion criteria. Therefore, 134 participants were enrolled and randomly assigned to PRF group or nerve block group using a randomization sequence generated by SAS software, which was coordinated by the central data center. The patients, pain physicians in charge of conducting the intervention treatment as well as the researcher responsible for follow-up evaluations were blinded to group allocation.

### PRF procedure

**Fig. 1:**
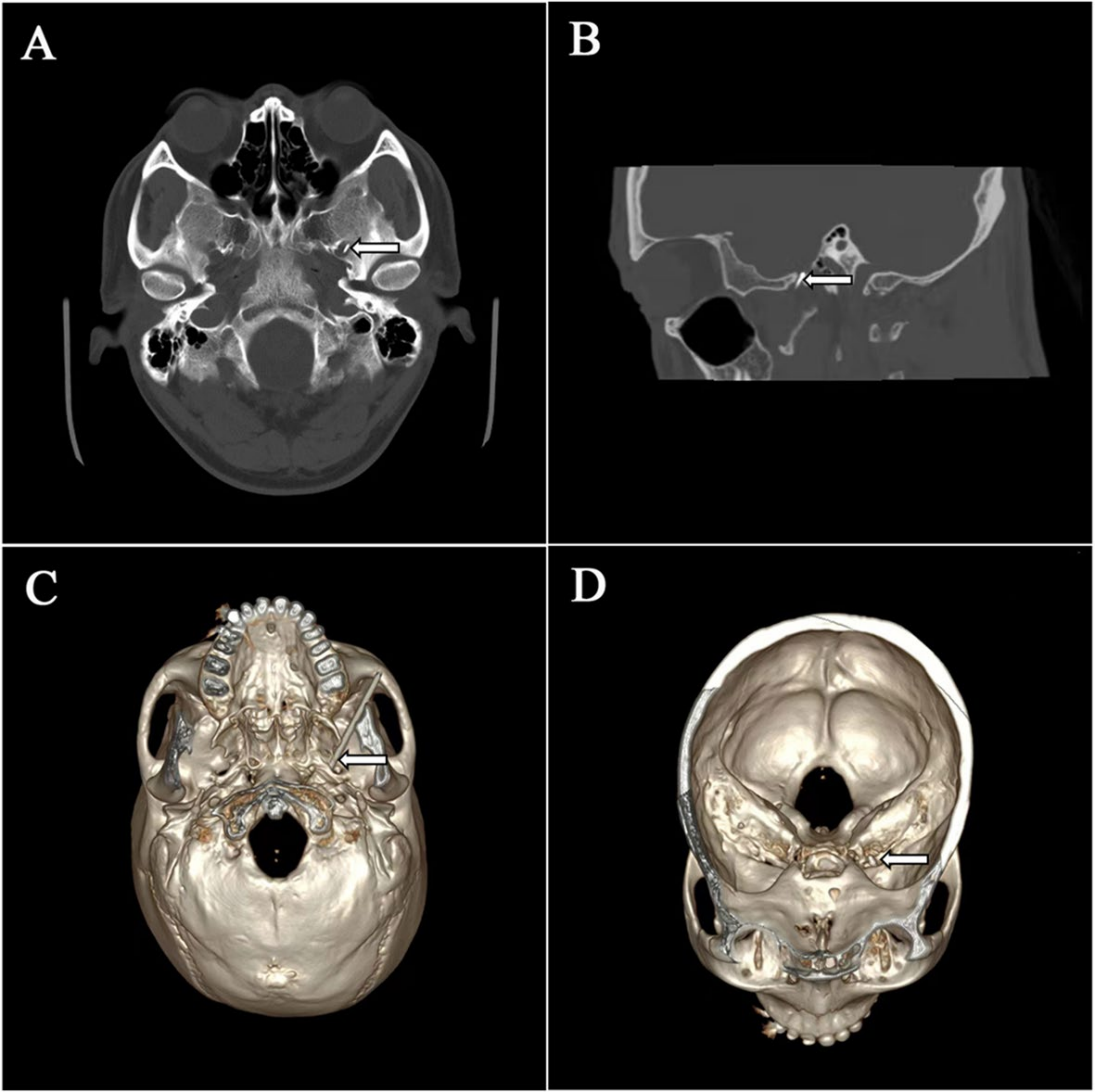
CT-guided puncture of the Gasserian ganglion. **A** Axial CT scan showing the needle (arrow) entering the foramen ovale. **B** Sagittal CT scan showing the needle (arrow) entering the foramen ovale. **C** 3D CT reconstruction of skull base view showing that the trocar entering the foramen ovale. **D** 3D CT reconstruction of interior skull base view showing that the trocar entering the foramen ovale

Patients were treated in the supine position on the computed tomography (CT) scanning table and were continuously monitored for blood pressure, heart rate, electrocardiogram and pulse oximetry. The negative plate of the PMG-230 Pain Management Generator (Baylis Medical Inc., Montreal, Canada) was attached to the patient’s back. The insertion point was approximately 3 cm beside the corner of the mouth on the affected side. After sterilization and local anesthesia with 1% lidocaine, a 21-gauge insulated needle trocar (10 cm, with a 5-mm active tip, Baylis Medical Inc., Montreal, Canada) was inserted and slowly advanced under the guidance of three-dimensional (3D) reconstruction using a thin-layer (2 mm/ layer) CT scan (SOMATOM SIEMENS Company, Munich, Germany) of the skull base until the trocar accurately punctured the foramen ovale. Then the stylet was removed, and the radiofrequency electrode (PMK-21-100, Baylis Medical Inc., Montreal, Canada) was inserted to test the resistance. Electrical stimulation was performed (sensory [50 Hz] and motor [2 Hz]) and the trocar position was adjusted corresponding to the patient’s sensation and movement to ensure accuracy of puncture location (Fig. 1). When the trocar reached the Gasserian ganglion, a randomized, sealed, opaque envelope was opened, and the patient was received high voltage PRF treatment or nerve block according to the concealed randomized treatment protocol. The parameters of PRF treatment and the specific operation process of nerve block were presented in our protocol published previously [19]. During the treatment period, the sound of the radiofrequency generator was turned off. Only the research nurse in charge of the allocation knew the treatment allocation.

### Data collection and follow-up

Preoperative data including age, gender, pain duration, pain laterality, divisions affected, preoperative BNI and preoperative carbamazepine dosage were recorded. Intraoperative parameters such as motor and sensory electrical stimulation voltage, output voltage, local tissue resistance and duration of procedure were recorded by the research nurse in charge of the allocation. The follow-up evaluations included time to take effect (the day on which that patients’ BNI score decreased to I- IIIb), postoperative short-term (postoperative day 1, week 1, week 2, month 1, month 2, month 3 and month 6) and long-term (year 1 and year 2) BNI scores [27], the incidence of adverse events (AEs) and the recovery condition.

### Outcomes

The primary outcome was the 1-year response rate. The degree of pain relief was.

evaluated as “excellent” (BNI pain score I-II), “good” (BNI pain score III), or “poor” (BNI pain score IV-V). The response rate was defined as [ patients with excellent and good pain relief (BNI pain score I- IIIb) ] /(total number of cases) × 100% after undergoing TN treatment. The secondary outcome included the response rates at day 1, week 1, week 2, month 1, month2, month 3, month 6, and year 2, intraoperative and postoperative AEs.

### Sample size

Based on a previous study [18] and our clinical experience, we assumed that the 1-year response rate of 1-time Gasserian ganglion nerve block treatment under CT guidance for TN patients was expected to be 40% (3). Our team had previously reported that the 1-year effective rate of high-voltage PRF on Gasserian ganglion under CT guidance to treat TN was 69% [24]. Therefore, a total of 60 patients in each arm would provide 90% power with a 2-sided type-I error of 0.05. Considering a dropout rate of 10%, the total sample size was estimated to be 134 patients (67 in each group).

#### Statistical analysis

The data were processed using SAS 9.4. Intention-to-treat(ITT)analysis and per-protocol (PP) analysis were performed. All data were tested for normality using Shapiro-Wilk test. Normal distribution variables were expressed as mean ± standard deviation (SD) and tested by student’s t test. Data without a normal distribution were expressed as median and interquartile range (IQR) and analyzed by Mann-Whitney U test. Categorical variables were presented as counts or percentages and tested by Chi-square test or Fisher’s exact test. In addition, comparison within groups was analyzed using paired t test or signed-rank test. Kaplan-Meier curves were used to show the cumulative proportion of recurrence-free survival.

Prespecified subgroup analyses included age (≤ 60 vs. >60 years), gender, disease duration(≤ 3 years or >3 years), laterality or branches affected(single branch vs. double or triple branches). Cox regression analysis was used to estimate the treatment-by-subgroup interaction effect. A *p*-value of ≤ 0.05 was statistically significant.

## Results

### Baseline characteristics

**Fig. 2:**
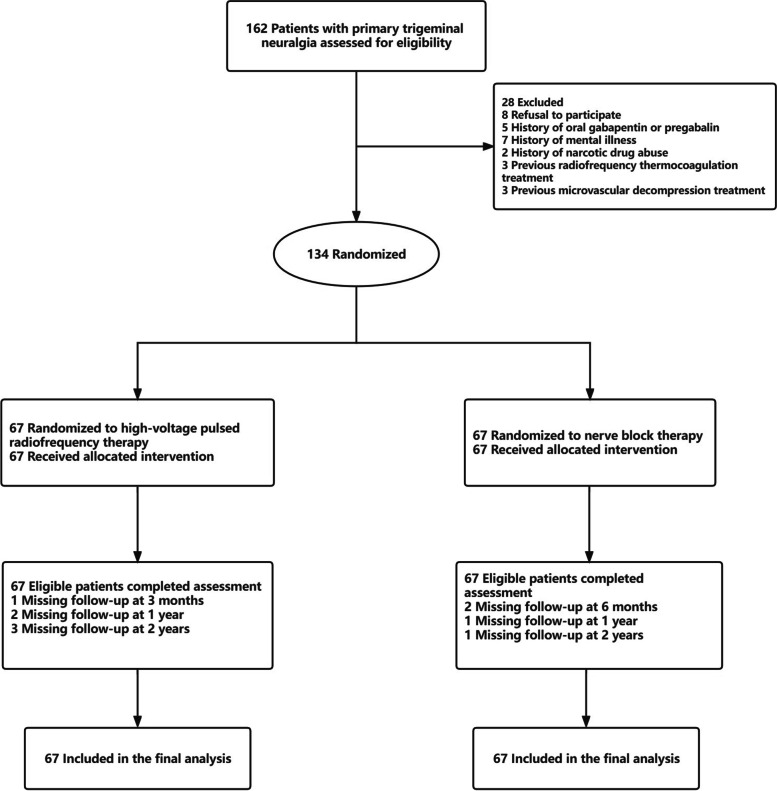
Flow diagram of the study populations

A total of 162 patients were screened at Beijing Tiantan Hospital, Beijing Friendship Hospital and Beijing Ditan Hospital between April 28th, 2017 and September 1st, 2019. Twenty-eight patients did not meet the inclusion criteria. As a result, 134 patients were randomly assigned to the PRF group and the nerve block group. One patient was lost to follow-up at 3 months in the PRF group, 2 patients were lost to follow-up at 6 months in the nerve block group. Two patients in the PRF group and 1 patient in the nerve block group were lost to follow-up 1-year postoperatively. Two years after the procedure, 3 patients in the PRF group and 1 patient in the nerve block group were out of reach by phone follow-up (Fig. 2). Missing data for 10 participants were imputed by the last observation carried forward method. Both groups were well matched regarding the baseline characteristics (Table 1).

**Table 1:** Baseline characteristics and intraoperative data of the patients

Variable	PRF group (*n* = 67)	Nerve block group (*n* = 67)	*P* value
Age (years)	58.24 ± 9.80	59.46 ± 11.69	0.203
Gender, male (%)	33 (49.3)	31(46.3)	0.729
Disease duration (months)	36 (24,45)	36 (24.5,60)	0.201
Laterality, right (%)	38 (56.7)	34 (50.7)	0.488
BNI score			
BNI IV	39 (58.2)	39 (58.2)	1.000
BNI V	28 (41.8)	28 (41.8)	
Dosage of Carbamazepine drugs (milligrams per day) Branch affected	500 (300–600)	500 (400–600)	0.194
V1, n (%)	1(1.5)	2 (3)	0.822
V2, n (%)	8(11.9)	7 (10.4)	
V3, n (%)	11(16.4)	9 (13.4)	
V1 + V2, n (%)	18(26.9)	13(19.4)	
V2 + V3, n (%)	25(37.3)	30 (44.8)	
V1 + V2 + V3, n (%)	4(6)	6(9)	
PRF output voltage (v)	77 ± 11.9		
2 Hz stimulating voltage (v)	0.1 (0.1,0.15)	0.1 (0.1,0.15)	0.758
50 Hz stimulating voltage(v)	0.1 (0.1,0.15)	0.1 (0.1,0.15)	0.481
Tissue resistance (Ω)	230 (219,244)	232 (219,249)	0.529
Surgery duration (min)	33 (32,36)	35(32,36)	0.342

Values are expressed as the means ± standard deviation, median (IQR), counts or percentages

*BNI *Counts or percentages, *PRF *Pulsed radiofrequency

### Primary outcome

For ITT analysis, the proportion of patients with positive response at 1-year after the procedure in the PRF group was significantly higher than that in the nerve block group (73.1% vs. 32.8%, RR,5.568; 95% CI,2.649–11.704; *p* < 0.001). For the primary outcome, the PP analysis (71.9% vs. 29.7%, RR, 6.053,95% CI, 2.818–13.001; *p* < 0.001;) showed a similar result as the ITT analysis.

### Secondary outcomes

In the ITT population, the postoperative response rates of the PRF group were higher than the nerve block group at week 2, month 1, month 2, month 3, month 6 and year 2 after the procedure (*p* = 0.002, *p* < 0.001, *p* <0.001, *p* < 0.001, *p* < 0.001, *p* < 0.001,respectively) (Table 2). PP analysis revealed that the response rate between the two groups at week 2, month 1, month 2, month 3, month 6 and year 2 after the procedure showed the same result as the ITT analysis (*p* = 0.002, *p* < 0.001, *p* < 0.001, *p* < 0.001, *p* < 0.001, *p* < 0.001, respectively) (see Supplement 1).

**Table 2:** Response rates after treatment (Intention-to-Treat Analysis)

Time point	PRF Group (*n* = 67)			Nerve block Group (*n* = 67)			RR (95%CI)	*P* Value
	Excellent pain relief	Good pain relief	Response Rate (%)	Excellent pain relief	Good pain relief	Response Rate (%)		
1 day	11	18	43.3	10	15	37.3	1.282(0.642–2.561)	0.481
1 week	15	18	49.3	10	16	38.8	1.531(0.771–3.040)	0.223
2 weeks	16	29	67.2	11	16	40.3	3.030(1.496–6.138)	0.002
1 month	18	32	74.6	11	15	38.8	4. 638(2.218–9.699)	< 0.001
2 months	18	32	74.6	11	14	37.3	4.941(2.357–10.359)	< 0.001
3 months	18	32	74.6	12	12	35.8	5.270(2.506–11.079)	< 0.001
6 months	19	30	73.1	10	14	35.8	4.877(2.337–10.178)	< 0.001
1 year	19	30	73.1	11	11	32.8	5.568(2.649–11.704)	< 0.001
2 years	20	29	73.1	10	12	32.8	5.568(2.649–11.704)	< 0.001

*PRF *Pulsed radiofrequency, *RR *Relative risk, *CI *Confidence interval

In the nerve block group, 24 patients attained immediate pain relief within 1 day after the procedure, 3 patients required a recovery period before achieving satisfactory pain relief. The median length of time to take effect in the nerve block group was 1 day (IQR,1–1 days). In the PRF group, 29 patients attained immediate pain relief within 1 day while 21 patients required a recovery period before achieving satisfactory pain relief. The median length of time to take effect in the PRF group was 1 day (IQR,1–11 days) which was significantly different from that in the nerve block group (*p* < 0.05).

**Fig. 3:**
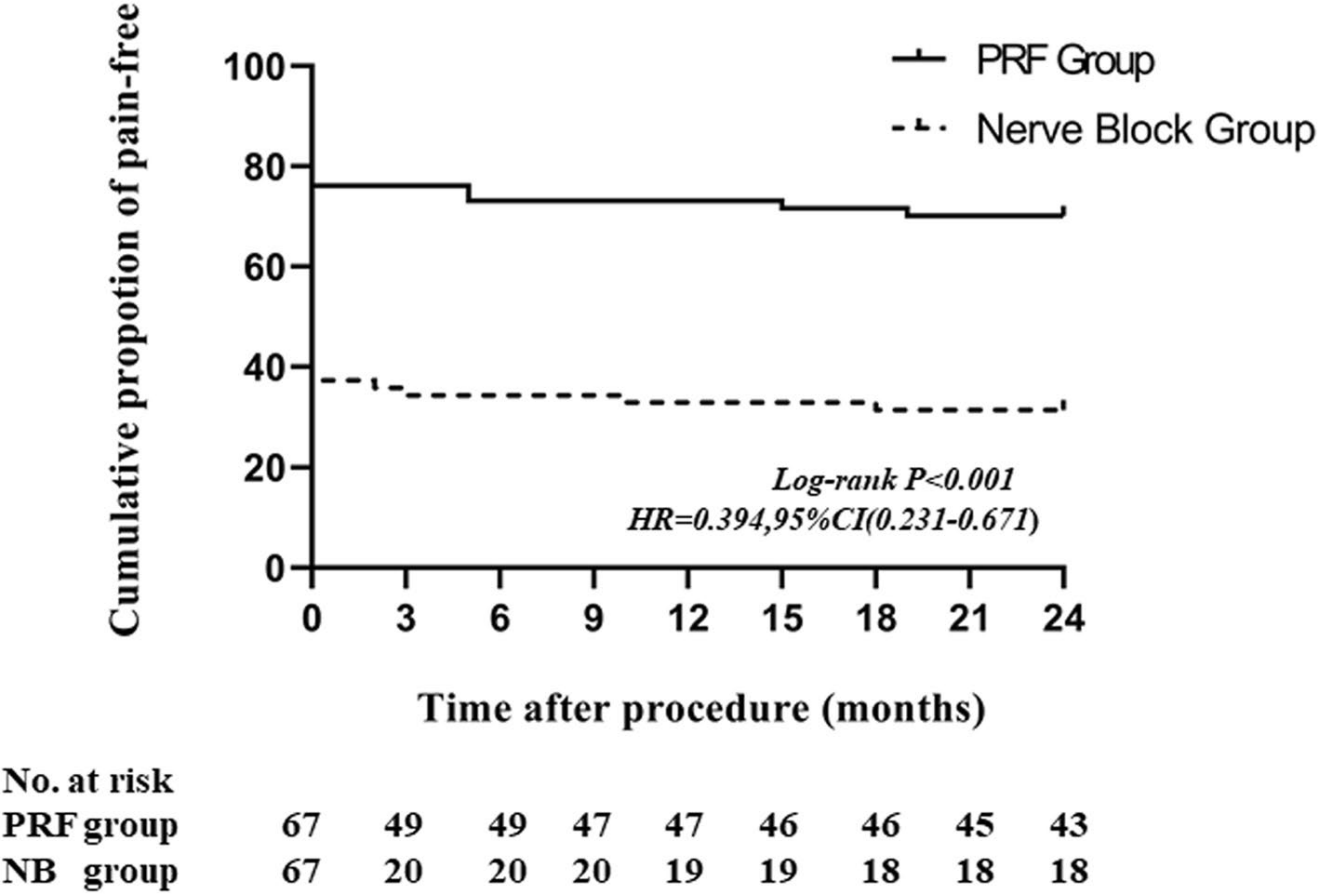
Kaplan-Meier recurrence-free survival curves for TN patients who underwent high-voltage PRF and nerve block treatment

The cumulative proportion of recurrence-free survival is shown as Kaplan–Meier curve (Fig. 3). After PRF treatment, the cumulative recurrence free survival was 76.1% at one month, 76.1% at 3 months, 73.1% at 6 months, 73.1% at 1 year and 70.1% at 2 years. After nerve block, the cumulative recurrence free survival was 37.3% at one month, 34.3% at 3 months, 34.3% at 6 months, 32.8% at 1 year and 32.3% at 2 years (HR,0.394; 95%CI, 0.231–0.671; *p* < 0.001).

**Fig. 4:**
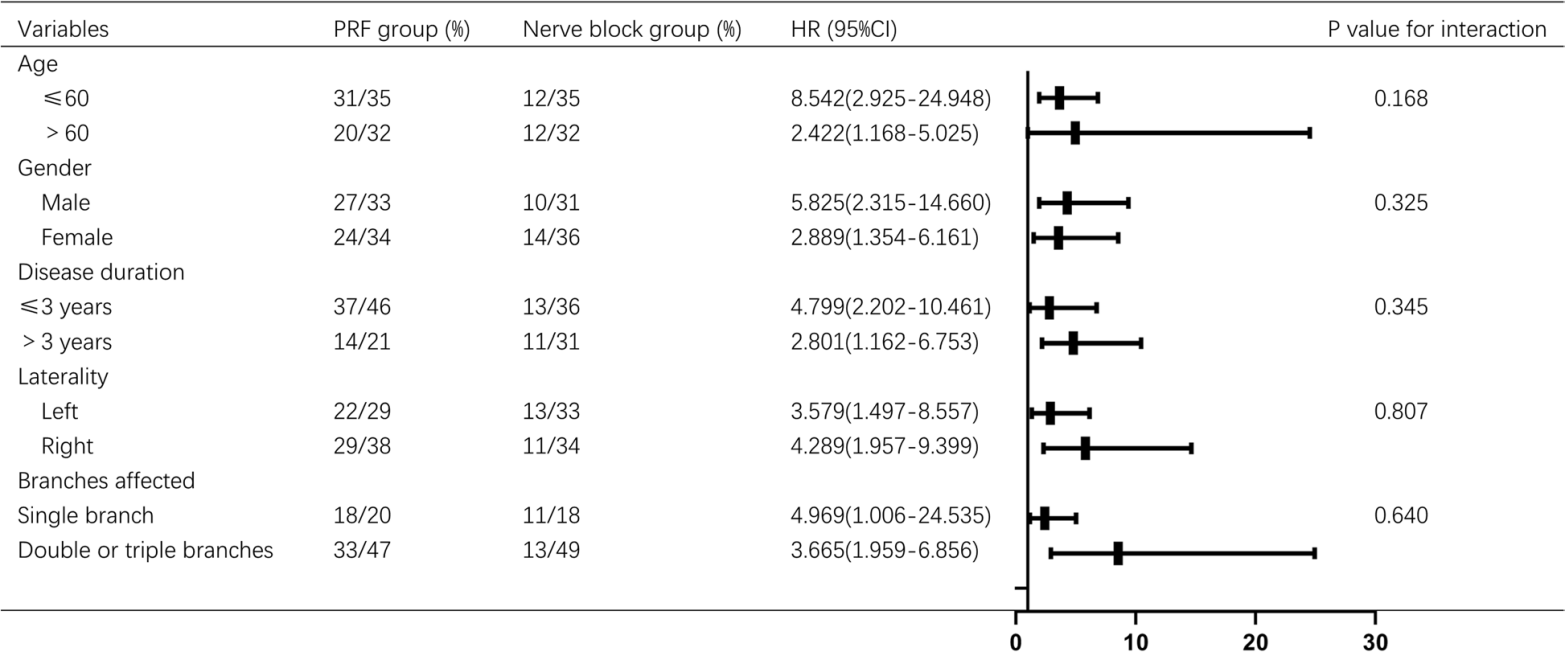
Subgroup analysis of the primary outcome in the prespecified subgroups

Subgroup analysis was based on the ITT population. There was no statistically significant difference in the interaction effect between high-voltage PRF and any of the 5 predefined subgroups (age, gender, disease duration, laterality or branches affected) (Fig. 4).

### Safety

The main adverse reaction during treatment was bradycardia while puncturing through the foramen ovale (heart rate < 60 beats/minute), which recovered spontaneously without treatment. Postoperative complications such as nausea or vomiting, postoperative dizziness, facial numbness and ecchymoma on the face occurred in a small number of patients in our study (Table 3). There was no difference between groups in the incidence of adverse events. However, patients with nausea or vomiting and dizziness gradually disappeared within 4 h after symptomatic treatment. The facial numbness improved within 1–2 months in the PRF group, and it improved within 2 weeks in the nerve block group. Facial ecchymoma gradually subsided within 2 weeks to 1 month. There was no infection, masseter weakness, intracranial hemorrhage, and any other adverse events during the follow-up period.

**Table 3:** Complications associated with PRF or nerve block treatment

	PRF group *n* = 67	Nerve block group *n* = 67	*P* value
Bradycardia(n, %)	3(4.5%)	1(1.5%)	0.619
Nausea or vomiting(n, %)	5(7.5%)	6(9.0%)	0.753
Postoperative dizziness(n, %)	4(6.0%)	5(7.5%)	1.000
Facial numbness(n, %)	7(10.4%)	3(4.5%)	0.189
Facial ecchymoma(n, %)	2(3.0%)	1(1.5%)	0.559

*PRF *Pulsed radiofrequency

## Discussion

In this prospective, multicenter, randomized, double-blind, controlled clinical study, we found that the postoperative one-year response rate (73.1%) of high-voltage PRF treatment was over twice more than that of nerve block (32.8%). And the cumulative recurrence-free survival in the PRF group was much higher than that in the nerve block group (HR, 0.394;95%CI, 0.231–0.671; *p* < 0.001). In our study, all the side effects were mild and gradually disappeared within 1–2 months and no significant differences were observed in complications associated with PRF or nerve block treatment (p > 0.05). As a result, our well-designed study demonstrates that, high-voltage PRF is an effective and safe treatment option for patients who respond poorly to drug treatment or experience intolerable medical side effects.

In 2013, our retrospective study revealed that the PRF output voltage in the effective group was significantly higher than that in the ineffective group (*p* < 0.05) [28]. A study published in 2013 also found different PRF output voltage since the same parameter were set due to different electronic resistance of each individual patient. And our subsequent single-center studies preliminarily verified that high-voltage PRF is an effective treatment option for TN patients [24, 25]. Similarly, in this study, the effective rate of high-voltage PRF treatment at week 2 was 67.2%. At 1 month after the procedure, the response rate increased to 76.1% and remained stable thereafter. 73.1% of patients with TN had a maintained satisfactory treatment effect 2 years after PRF treatment, which was significantly higher than that in the nerve block group (*p* < 0.001) and is consistent with our previous study (78.6%) [25]. The 1-year response rate of 1-time nerve block treatment with local anesthetic and steroid for TN in previous studies was about 25–40% [16, 29, 30], which was similar to our study (35.8%). However, the effective treatment in our previous study was defined as the reduction of Numeric Rating Scales (NRS) score by ≥ 50%; we used the BNI scale in this study as it involves both pain intensity as well as dosage of antiepileptic drugs and can therefore achieve a more comprehensive assessment of the therapeutic effect. Moreover, this study was a multicenter, randomized, double-blind trial which could provide a higher level of evidence for TN therapy. Our findings are encouraging, as more than two-thirds of TN patients receiving high voltage PRF attained satisfactory pain relief. This can provide a novel micro-destructive treatment option for patients who had failed to respond to conservative drugs therapy and have to undergo a more invasive or neuro-destructive surgical therapy.

In our study, there was a significant difference in the time to take effect between the two groups. Most patients in the nerve block group achieved immediate pain relief within 1 day after the procedure, while more than 40% patients in the PRF group required a recovery period before achieving satisfactory pain relief. About 10% patients in the PRF group did not experience immediate effective pain relief until 3 weeks postoperatively. These results are similar to the ones from our previous studies regarding PRF treatment for TN patients [24, 28, 31–34]. The reason for this delayed effect could be because PRF treatment can cause plastic changes in pain transmission pathways and lead to slow neuromodulation, which would take a longer time to achieve sufficient pain relief in certain patients. As a result, individual variability in pain response after PRF treatment should be taken into consideration by pain physicians, such as continued prescription of adequate anti-epileptic drugs before achieving satisfactory analgesic efficacy.

The main complications associated with PRF or nerve block treatment included bradycardia, nausea or vomiting, postoperative dizziness, facial numbness and facial ecchymoma. A total of 4.5% patients in the PRF group and 1.5% patient in the nerve block group experienced bradycardia during puncture. Although spontaneous recovery occurred in all cases without treatment, this occurrence of bradycardia further emphasizes the importance of monitoring vital signs during the procedure. There was a small number of patients with postoperative nausea or vomiting. Fortunately, these symptoms gradually improved after supportive treatment. Whether prophylactic administration of antiemetics can reduce the incidence of postoperative nausea or vomiting has not been reported. There were a few patients suffering from dizziness and the symptom disappeared within a few hours. The exact mechanism leading to dizziness and effective preventive measures to reduce their occurrence remains to be studied further. These complications suggest that the patients’ postoperative vital signs must be closely monitored and managed until patients can be safely discharged. Although there was no significant difference in the incidence of facial numbness between the two groups, the recovery time of patients in the nerve block group was shorter than that in the PRF group. We speculated that facial numbness in the nerve block group was due to nerve injury caused by puncture. However, numbness in PRF group was related to the pathological changes of nerve tissue caused by high electric field as well as puncture injury, which needed a longer recovery time. 3% patients in the PRF group and 1% patients in the nerve block group experienced facial ecchymoma due to the injury of facial vasculature caused during puncture. Fortunately, facial ecchymoma could be absorbed spontaneously within 1 month. However, the incidence of complications was similar between the two groups, which revealed that high-voltage PRF is a safe, minimally invasive technique for the treatment of TN.

Up to now, there is no standard optimal parameters for PRF therapy. Similar to our previous study [24], the output voltage of 77 ± 11.9 V which could achieve satisfactory analgesic efficacy without serious complications. The exposure time of PRF current in this study was 360 s, which is in accordance with Jia et al.’s study [34]. Some researchers proposed that increasing the treatment time is associated with more effective pain relief effect [35, 36]. High-voltage, long-duration PRF has been successfully performed for the treatment of discogenic pain [37], pudendal neuralgia [36, 38] as well as acute/subacute zoster-related trigeminal neuralgia [39]. Nevertheless, there is a paucity of research to evaluate whether increasing the exposure time could further improve the therapeutic effect of high-voltage PRF for the treatment of TN. As a result, a standard optimal parameter to obtain the best analgesic effect of PRF deserves further investigation.

In the past, radiofrequency treatment for TN patients was usually performed under C-arm fluoroscopic guidance which is associated with tissue injury caused by inaccurate puncture [23, 40, 41]. In this study, all patients underwent PRF treatment or nerve block under the guidance of 3D-CT which could ensure the puncture accuracy and prevent severe complications associated with inaccurate puncture such as carotid-cavernous fistula or cerebrospinal fluid leakage. Puncture success rate in our trial was 100%, and no puncture-related serious side effects were observed. However, patients had to inevitably be exposed to radiation when receiving treatment under guidance of 3D-CT. There are many other navigation techniques currently available for accurate cannulation of the foramen ovale. Neuronavigation techniques such as electromagnetic navigation, optoelectronic tracking systems, 3D templates, etc. have gained popularity recently due to their preciseness and lack of risk of radiation exposure to both patients and hospital staff. However, the probes and tracers of the electromagnetic navigation devices are not only disposable, but also expensive and are easily distorted by external magnetic signals [42]. Moreover, “an uninterrupted line-of-sight” is necessary for optoelectronic tracking systems which can result in difficult cannulation near reflective spheres [43]. A recently published literature review and meta-analysis by Wang et al. reported that the one puncture success rates of the stereotactic technique, 3D template and neuronavigation were 99%, 93% and 69% ,respectively, for radiofrequency thermocoagulation with different guidance techniques for trigeminal neuralgia; whereas, CT navigation achieved one-puncture success rates of 64.3 − 100%. However, no two methods were compared in a single study included in this review, leaving the success rate at the discretion of the surgeon [44]. Ultrasonography seems to be a brief operational modality which could provide visualization of the soft tissues and vascular structures in real time without exposure to radiation energy [45]. In recent years, ultrasound-guided interventional procedures have been successfully performed in the treatment of several types of neuropathic pain (NP) [38, 46–50]. Up to now, there is a lack of study on ultrasound guided interventional treatment of Gasserian ganglion for TN patients that needs to be further explored. With improvements in ultrasonography, prospective, randomized controlled clinical trials are needed to explore in the future, to determine whether ultrasonography is an ideal imaging tool for the guidance of PRF treatment in TN patients.

Our study has several limitations that need to be addressed in future trials. First, our follow-up period was only 2 years, longer follow-up duration (such as 10 years) is required to confirm long-term efficacy of PRF therapy in TN patients. Secondly, in order to answer whether a simple and economical nerve block therapy (using steroids and local anesthetics) or pulsed radiofrequency is the appropriate treatment of choice, we compared pulsed radiofrequency with nerve block and investigated whether pulsed radiofrequency can become a choice before surgical treatment with ineffective drug therapy. The most optimal parameters of PRF such as treatment duration, output voltage or temperature should be further investigated to achieve the most satisfactory analgesic effect with maximum safety. Moreover, a multicentric RCT comparing PRF of 45 V and PRF of higher output voltage could have provided a more valuable insight. Furthermore, there was a lack of recording and analysis of different pain symptoms including allodynia, stabbing pain or continuous ache or burn. Last but not least, follow up regarding patients’ QoL was not conducted in our study, which would require further improvement.

## Conclusion

To the best of our knowledge, this is the first prospective, multicenter, randomized, double blinded controlled clinical study providing strong evidence that high-voltage PRF can achieve more satisfactory efficacy than nerve block for TN therapy. This effective, minimally invasive strategy could be the preferred choice of intervention prior to more invasive, surgical, or neuro-destructive treatment options for TN patients who respond poorly to medical treatment.

## Supplementary Information

**Additional file 1.**

## Data Availability

The datasets of the current study are available from the corresponding authors upon reasonable request.

## Abbreviations

TN: Trigeminal neuralgia
QoL: Quality of life
MVD: Microvascular decompression
BC: Balloon compression
PRF: Pulsed radiofrequency
GCP: Good Clinical Practice
IRB: Institutional Review Board
ICHD-3: International Classification of Headache Disorders 3rd Edition
BNI: Barrow Neurological Institute
CT: Computed tomography
3D: Three-dimensional
ITT: Intention-to-treat
PP: Per-protocol
SD: Standard deviation
IQR: Interquartile range
NRS: Numeric Rating Scales
NP: Neuropathic pain
