# Supplementary material for: Effectiveness and safety of high-voltage pulsed radiofrequency to treat patients with primary trigeminal neuralgia: a multicenter, randomized, double-blind, controlled study
Source: J Headache Pain. 2023 Jul 18;24(1):91. doi: 10.1186/s10194-023-01629-7 (PMC10353218; doi:10.1186/s10194-023-01629-7)
Supplement: Supplementary file 1 — Additional file 1. [file 10194_2023_1629_MOESM1_ESM.docx]

Supplement 1. Response rates After Treatment (Per-Protocol Analysis)

| Time point | PRF Group (n=67) | | | Nerve block Group (n=67) | | | RR  (95%CI) | P Value |
| --- | --- | --- | --- | --- | --- | --- | --- | --- |
|  | Excellent pain relief | Good pain relief | Response Rate (%) | Excellent pain relief | Good pain relief | Response Rate (%) |  |  |
| 1 day | 11 | 18 | 43.3 | 10 | 15 | 37.3 | 1.282(0.642-2.561) | 0.481 |
| 1 week | 15 | 18 | 49.3 | 10 | 16 | 38.8 | 1.531(0.771-3.040) | 0.223 |
| 2 weeks | 16 | 29 | 67.2 | 11 | 16 | 40.3 | 3.030(1.496-6.138) | 0.002 |
| 1 month | 18 | 32 | 74.6 | 11 | 15 | 38.8 | 4. 638(2.218-9.699) | ＜0.001 |
| 2 months | 18 | 32 | 74.6 | 11 | 14 | 37.3 | 4.941(2.357-10.359) | ＜0.001 |
| 3 months | 18 | 31 | 74.2 | 12 | 12 | 35.8 | 5.164(2.454-10.869) | ＜0.001 |
| 6 months | 19 | 29 | 72.7 | 10 | 12 | 33.8 | 5.212(2.470-10.996) | ＜0.001 |
| 1 year | 19 | 27 | 71.9 | 11 | 8 | 29.7 | 6.053(2.818-13.001) | ＜0.001 |
| 2 years | 20 | 23 | 70.5 | 10 | 8 | 28.6 | 5.972(2.750-12.971) | ＜0.001 |
